# Mir106b-25 and Mir17-92 Are Crucially Involved in the Development of Experimental Neuroinflammation

**DOI:** 10.3389/fneur.2020.00912

**Published:** 2020-08-21

**Authors:** Annamaria Finardi, Martina Diceglie, Luca Carbone, Caterina Arnò, Alessandra Mandelli, Giuseppe De Santis, Maya Fedeli, Paolo Dellabona, Giulia Casorati, Roberto Furlan

**Affiliations:** ^1^Clinical Neuroimmunology Unit, Division of Neuroscience, Institute of Experimental Neurology, San Raffaele Scientific Institute, Milan, Italy; ^2^Experimental Immunology Unit, Division of Immunology, Transplantation and Infectious Diseases, San Raffaele Scientific Institute, Milan, Italy

**Keywords:** MicroRNAs, experimental autoimmune encephalomyelitis, multiple sclerosis, IL-17, Th17, miR106b-25, miR17-92

## Abstract

MicroRNAs (miRNAs) are single-stranded RNA that have key roles in the development of the immune system and are involved in the pathogenesis of various autoimmune diseases. We previously demonstrated that two members of the miR106b-25 cluster and the miR17-92 paralog cluster were upregulated in T regulatory cells from multiple sclerosis (MS) patients. The aim of the present work was to clarify the impact of miR106b-25 and miR17-92 clusters in MS pathogenesis. Here, we show that the mice lacking miR17-92 specifically in CD4^+^ T cells or both total miR106b-25 and miR17-92 in CD4^+^ T cells (double knockout) are protected from Experimental Autoimmune Encephalomyelitis (EAE) development while depletion of miR106b-25 only does not influence EAE susceptibility. We suggest that the absence of miR106b does not protect mice because of a mechanism of compensation of miR17-92 clusters. Moreover, the decrease of neuroinflammation was found to be associated with a significant downregulation of pro-inflammatory cytokines (GM-CSF, IFNγ, and IL-17) in the spinal cord of double knockout EAE mice and a reduction of Th17 inflammatory cells. These results elucidate the effect of miR106b-25 and miR17-92 deletion in MS pathogenesis and suggest that their targeted inhibition may have therapeutic effect on disease course.

## Introduction

miRNAs take part in the regulation of immune processes not only during innate and adaptive immune system development but also in its homeostasis, as well as in disease, regulating immune cell functions (1–3) and cytokine expression (4, 5). We analyzed in the past, the miRNA expression profile in regulatory T cells (Tregs) from relapsing-remitting multiple sclerosis patients (RR-MS) (6). We identified 23 microRNAs differentially expressed in MS Tregs as compared to Tregs from healthy controls. In particular, we found two members of the miR106b-25 cluster (miR93, miR106b) and two members of the miR17-92 paralog cluster (miR-19a and miR-19b) upregulated in Treg cells from MS patients (6). Further, the over-expression of miR17-92 in lymphocytes induces lymphoproliferative disease and autoimmunity in mice (7).

The miR106b-25 cluster (in particular miR-25 and miR-106b) over-expression can silence two important effectors of the TGF-β signaling pathway: the cell cycle inhibitor CDKN1A (p21) and the pro-apoptotic gene BCL2L11 (BIM) (8). Several results suggest that miR 106b-25 and miR17-92 clusters cooperate in inactivating the TGF-β pathway (8). TGF-β is an important immunomodulatory cytokine involved in the maintenance of self-tolerance and T cell homeostasis (9). miR17-92 and miR106b-25 clusters are ubiquitously expressed (10). During lymphocyte development, miR17-92 miRNAs are highly expressed in progenitor cells, expression levels decreasing 2- to 3-fold upon lymphocyte maturation (10, 11). miR17-92 regulates B- and T-cell development and its absence results in enhanced proliferation and survival of B- and T-cells. This is apparently due to increased expression of two target genes, namely the apoptosis facilitator BCL2L11 (BIM) and the tumor enhancer phosphatase and tensin homology (PTEN). PTEN is an inhibitor of the PI3K pathway promoting cell cycle progression and inhibiting apoptosis by negatively regulating the transcription of BIM (11). Members of this cluster clearly cooperate in the context of TGF-β signaling. miR-17 and miR-20a, for example, target directly the TGF-β receptor II (TGFBRII), while miR-18a targets other two members of the TGF-β signaling pathway, namely Smad2 and Smad4 (12–14). BIM and p21 are two mediators of the effects downstream to TGF-β activation. The host gene for the miR-106a/25 cluster, Mcm7, is down regulated during endoplasmic reticulum related stress, by the activation of transcription factor 4 (Atf4) and nuclear factor-erythroid-2-related factor 2 (Nrf2). This causes down-regulation of miR-106b/25 and repression of BCL2L11, and consequently apoptosis (7). Functional redundancy of homologous miRNAs having similar expression patterns may explain the lack of an obvious phenotype in mice deficient for miR106b-25, whose function is apparently largely compensated by miR17-92. Constitutive deletion of the two clusters, on the other hand, is embryonically lethal (10). We induced experimental autoimmune encephalomyelitis (EAE) in mice constitutively deleted for the miR106b-25 cluster, in mice lacking the miR17-92 in CD4^+^ T cells, and in mice with both mutations, to try to dissect the contribution of these miRNA families to neuroinflammation.

## Results

### Mice Lacking Mir17-92 or Both Mir17-92 and Mir106b Are Protected From Clinical Signs of Eae

**Figure 1:**
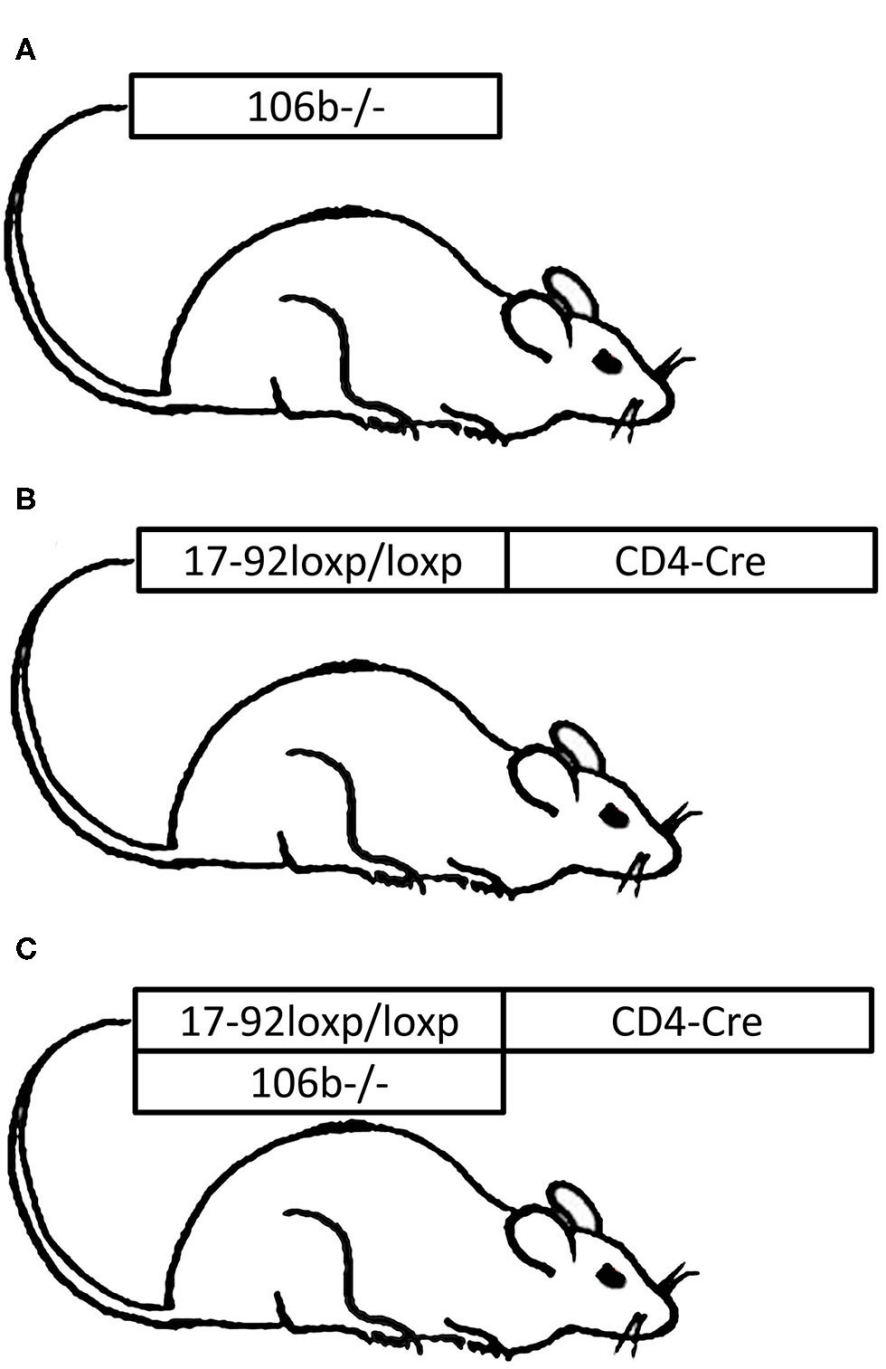
Graphic representation of the three mutant strains used for the EAE experiments. Experimental Autoimmune Encephalomyelitis (EAE) was induced in mice constitutively deleted for **(A)** miR106b-25 cluster (106b^−/−^ mouse), **(B)** miR17-92 in CD4+ T cells (17-92^−/−^ mouse), or in mice with **(C)** both mutations (106b^−/−^17-92^−/−^ mouse). miR17-92 was conditionally deleted only in CD4+ T cells because mice deficient for miR-17-92 die shortly after birth (10).

**Figure 2:**
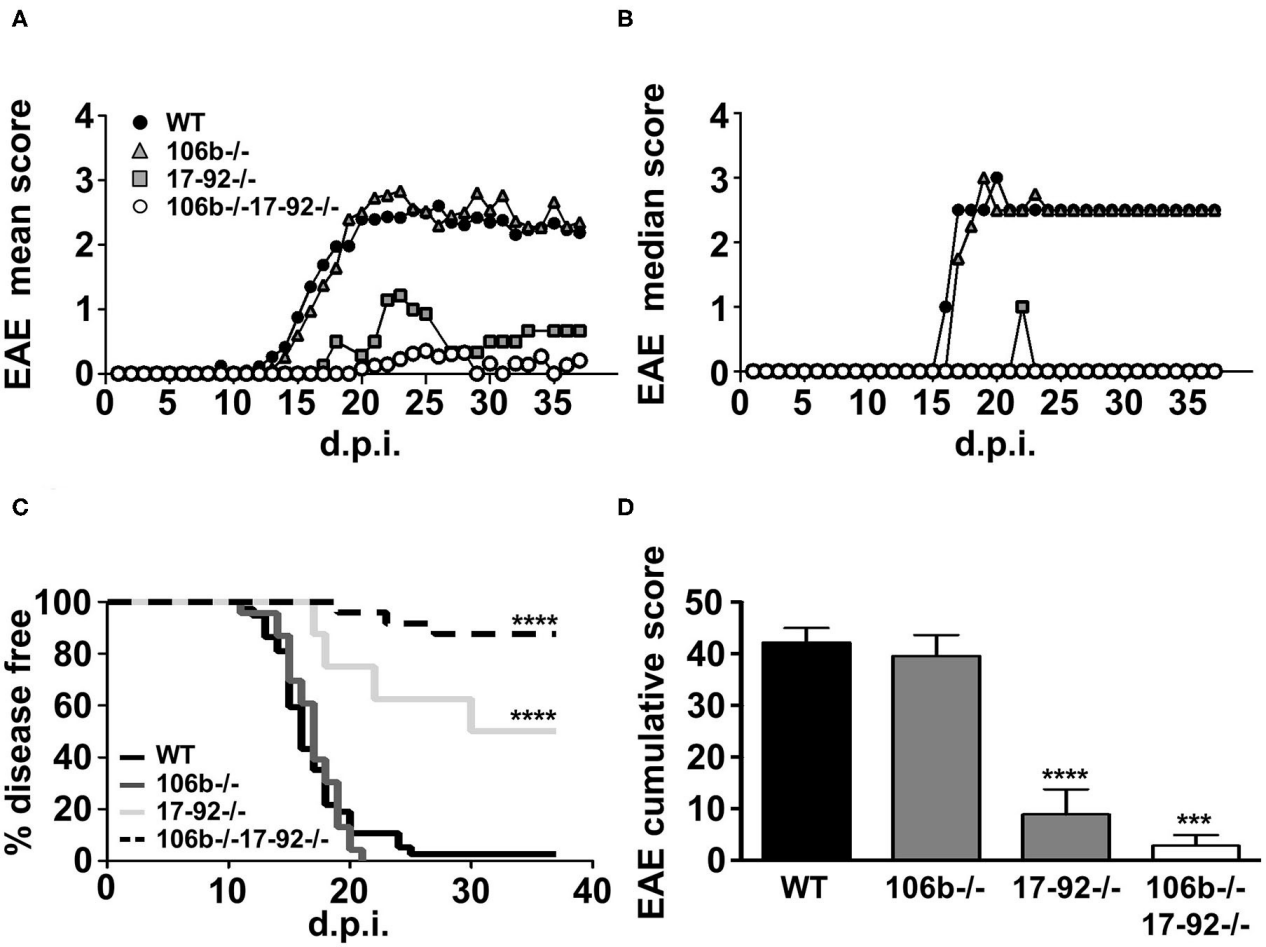
Mice lacking miR17-92 or both miR17-92 and miR106b are protected from clinical signs of EAE. WT (*n* = 41), 106b^−/−^ (*n* = 29), 17-92^−/−^ (*n* = 8), and 106b^−/−^17-92^−/−^ (*n* = 25) mice were immunized to induce EAE. Clinical signs of EAE were monitored daily until day 37 post-immunization. Mean **(A)** and median **(B)** clinical score and disease incidence **(C)** were assessed for each group. The Log-Rank (Mantel Cox test) was used for the comparison of EAE incidence rates between the different groups. EAE was evaluated as cumulative score using Mann Whitney test **(D)**. Error bars indicate mean ± SEM. ***P ≤ 0.001 and ****P ≤ 0.0001.

We used the mutant strains depicted in Figure 1. Since mutant mice colonies have been maintained in heterozygosity, littermates have been used as appropriate WT controls. All mice were on the C57BL/6 background and we therefore induced EAE by MOG_35−55_ immunization. As shown in Figure 2, the absence of both miR106b and miR17-92, almost abolished susceptibility to EAE, with 90% of mice remaining disease free, and 10% displaying very mild and transient clinical signs (Figures 2A–D). The absence of miR17-92 only resulted in a mild disease with 50% incidence, while mice lacking only miR106b did not differ from wild type control mice, displaying full-blown EAE (Figures 2A–D).

### Mice Lacking Mir17-92 or Both Mir17-92 and Mir106b Are Protected From Pathological Signs of Eae

**Figure 3:**
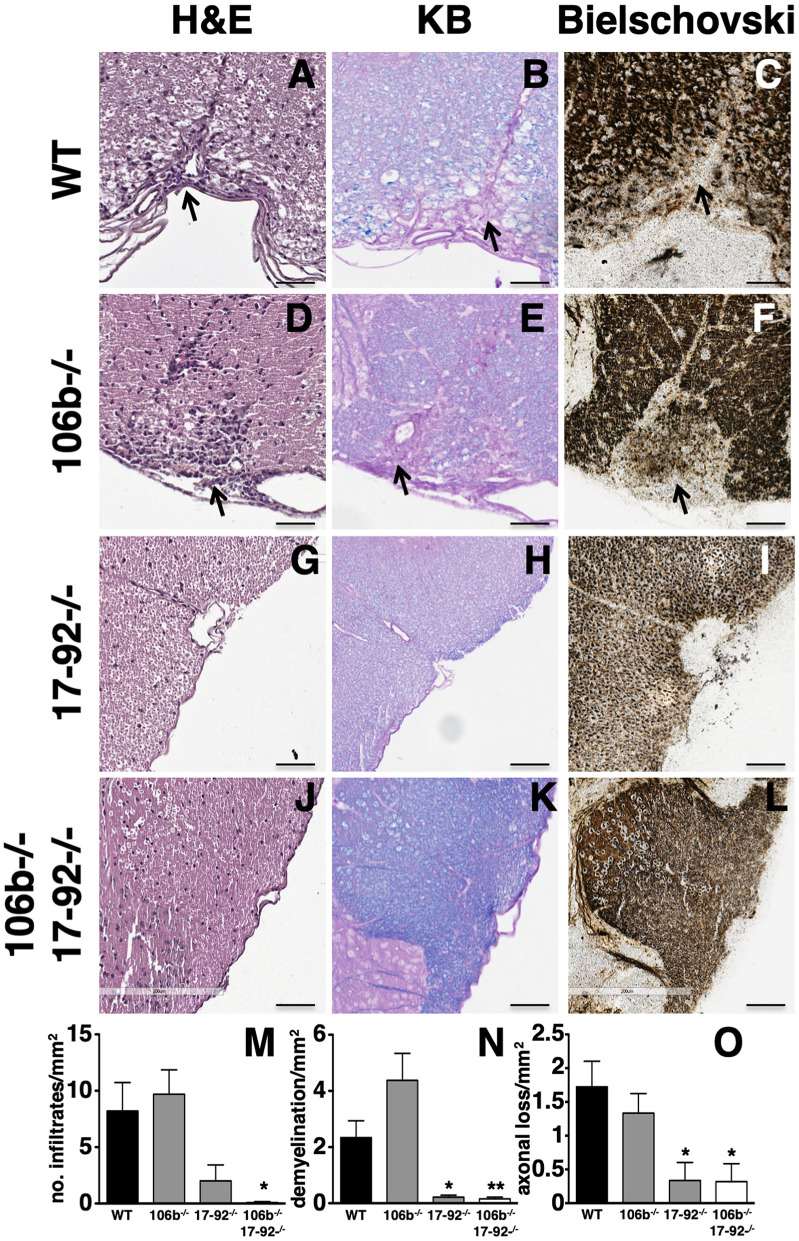
Lack of miR17-92 or both miR17-92 and miR106b protects mice from pathological signs of EAE. Neuropathological analysis of infiltrates (Hematoxylin and Eosin; **A,D,G,J**), demyelination (Kluver Barrera; **B,E,H,K**), and axonal loss (Bielschowsky; **C,F,I,L**) in the spinal cord. Scale bar = 100 μm. Deletion of miR17-92 or both miR17-92 and miR106b is associated to a significant decrease of number of infiltrates **(M)**, demyelination **(N)**, and axonal damage **(O)**. Data are shown as mean ± SEM. Statistical significance was determined by Mann Whitney test. *P ≤ 0.05 and **P ≤ 0.01.

Clinical findings in mice lacking miR17-92, or miR17-92 and miR106b, are paralleled by neuropathological findings. Indeed, the number of inflammatory infiltrates (Figures 3A,D,G,J), demyelination (Figures 3B,E,H,K) and axonal damage (Figures 3C,F,I,L) in the spinal cord were significantly decreased (Figures 3M–O) in mice lacking miR17-92 or both miR17-92 and miR106b, while in mice lacking only miR106b tissue damage was similar to that in control mice (Figures 3D–F,M–O and Figure S1).

### Absence of Mir106b Is Compensated by Mir17-92 Overexpression but Not Viceversa

**Figure 4:**
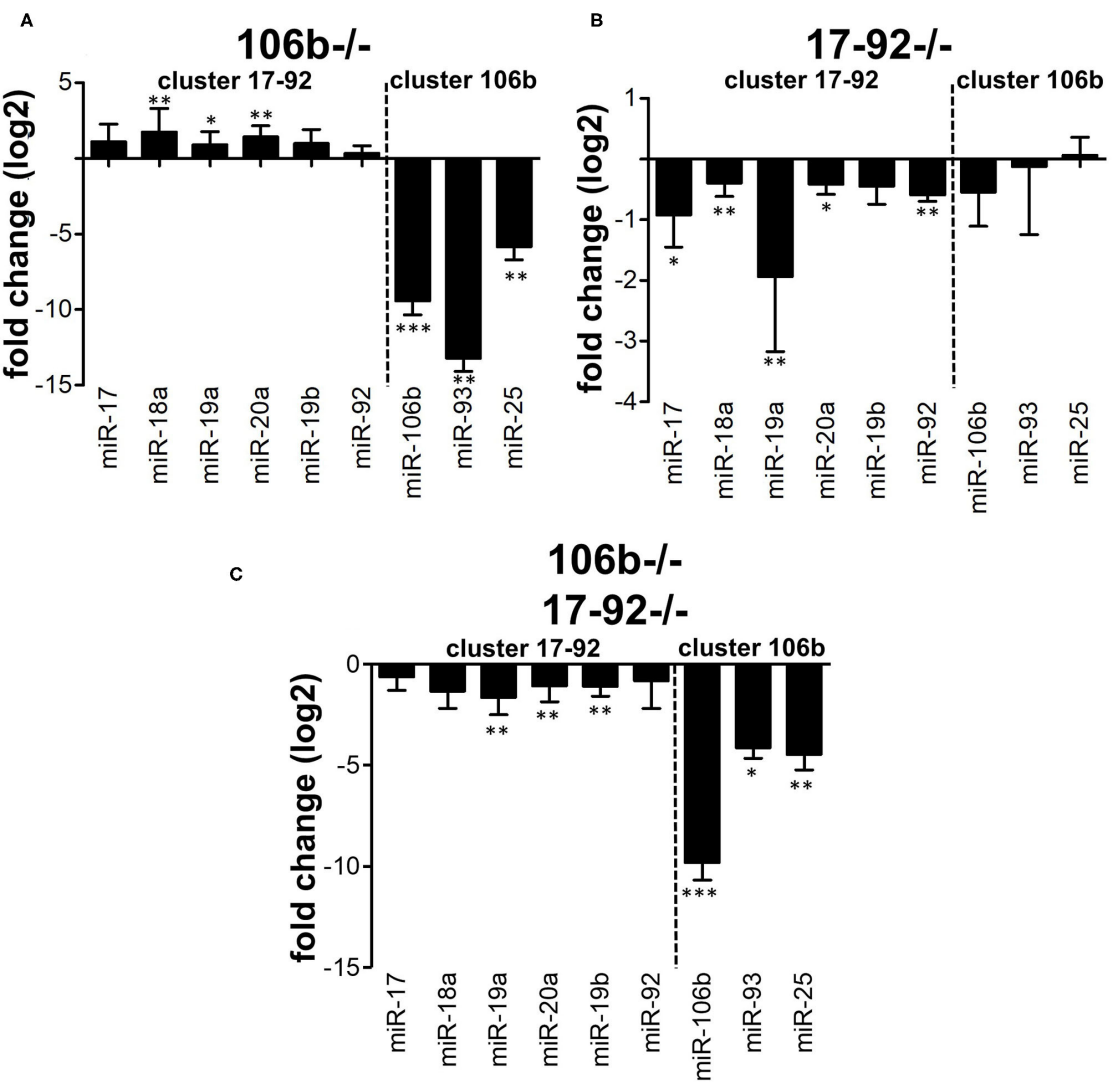
Compensation between miR106b and miR17-92 family clusters. Relative expression (log_2_ fold change vs. WT mice) of the 17-92 and 106b family clusters was measured by real time RT-PCR in CD4+ T cells isolated from the spleen of naive 106b^−/−^ **(A)**, 17-92^−/−^ **(B)**, and 106b^−/−^17-92^−/−^ **(C)** mice. Data are shown as mean ± SEM. miRNA of the two different clusters (miR 106b-25 and miR 17-92 clusters) are divided by a dotted line. * <0.05; ** <0.01; *** <0.001 Mann-Whitney.

We hypothesized that the absence of any alteration of the disease phenotype in miR106b^−/−^ mice might be due to compensation by the miR17-92 cluster. This may occur functionally or also due to overexpression. We therefore analyzed, by RT-PCR, the expression of the miRNAs from the two clusters in CD4^+^ T cells purified from the spleen of naïve mice. We found that miRNAs from the miR17-92 cluster were up-regulated in mice lacking the miR106b cluster (Figure 4A), while the opposite did not occur (Figure 4B). miRNAs levels in double deletion mutant mice are shown for comparison (Figure 4C). When we measured mRNA levels of classical targets for miR17-92 and miR106b however, namely p21, BIM, and STAT3, in brain, spinal cord, lymph nodes, and spleen, we did not find any significant difference, with the exception of a slight decrease of BIM and STAT3 in the spinal cord of EAE mice deleted for miR17-92 and miR106b (Figure S2).

### Pro-inflammatory Cytokine Expression Is Decreased in Eae Mice Lacking Mir17-92 and Mir106b

**Figure 5:**
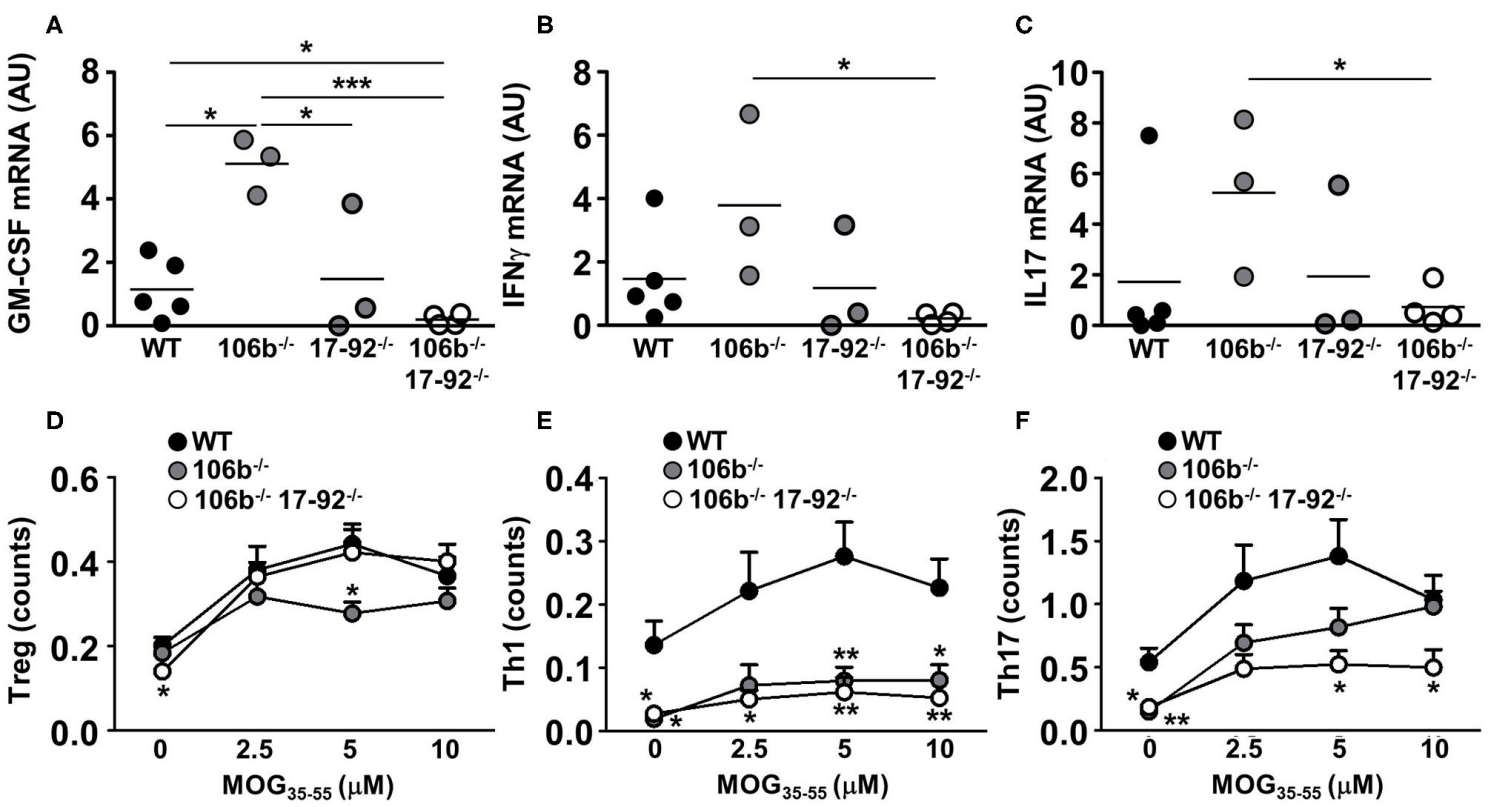
Reduction of pro-inflammatory cytokines in mice lacking miR17-92 and miR106b. **(A–C)** mRNA levels of GM-CSF, IFNγ, and IL17 were measured in the spinal cord by real time RT-PCR. Data are shown as arbitrary units (AU). **(D–F)** Total splenocytes were isolated from mice spleen (*n* = 5 for each group) 37 days after EAE induction. Cells were re-stimulated for 3 days with increasing amounts of MOG_35−55_ and the absolute count of Treg, Th1, and Th17 cells (% singlets on 20,000 cells X % Treg or Th1 or Th17 on singlets/100) was measured by flow cytometry. Statistical significance was determined by Mann Whitney test. *P ≤ 0.05, **P ≤ 0.01, and ***P ≤ 0.001.

We next examined the expression of cytokines known to drive neuroinflammation in the spinal cord of EAE mice. We found that GM-CSF (Figure 5A), IFNγ (Figure 5B), and IL17 (Figure 5C) mRNA levels are significantly reduced in mice deleted for miR17-92 and miR106b as compared to mice lacking only miR106b or WT EAE mice. Mice lacking only miR17-92 display a non-significant decrease of these mRNAs, coherent with the disease phenotype which is in between the double deletion mutants and the miR106b and the WT EAE mice.

### Th17 Cells Are Decreased in Mir17-92 Knock Out Mice

The reduction of pro-inflammatory cytokines may merely reflect the decrease of inflammatory cell infiltration in the CNS and the consequent decrease of neuroinflammation. To investigate what cell type is more affected in its development by the absence of miR17-92 and miR106b we therefore induced EAE in mice lacking miR106b, or miR106b and miR17-92, and WT mice and harvested spleens 37 days later. We isolated total splenocytes and re-stimulated them with the nominal antigen at escalating doses, and by intracellular staining followed by flow cytometry we measured the absolute numbers of Tregs, Th1, and Th17 cells. We found no differences in Tregs (Figure 5D), a significant decrease of Th1 cells in both single miR106b and miR106b and miR17-92 double deletion mutant mice as compared to WT mice (Figure 5E), but a significant decrease in Th17 cells specifically in miR106b and miR17-92 double deletion mutant mice (Figure 5E).

## Discussion

miRNAs have been demonstrated to regulate several processes in the development and function of the immune system and the alteration of miRNA homeostasis was shown to lead to the dysfunction of immune responses causing autoimmune diseases such as MS.

In our previous studies, we analyzed miRNA expression profile in Tregs from relapsing remitting MS patients and we identified two members of the miR 106b-25 cluster (miR93, miR 106b) and two members of the miR17-92 paralog cluster (miR 19a and miR 19b) as increased in Tregs from relapsing remitting MS patients compared to healthy controls (6).

The influence of miR 17-92 cluster in Treg cell differentiation was demonstrated in MS also in a study of miRNA profiling. Nineteen miRNAs (including miR18a that belongs to miR 17-92 cluster) were found differentially expressed in naive CD4 T cells multiple sclerosis patients and predicted to target TGFβ. These miRNAs negatively regulated the TGFβ pathway (TGFBR1 and SMAD4 were significantly reduced in patients with multiple sclerosis), resulting in a decreased capacity of naive CD4 T cells to generate regulatory T cells (15).

Moreover, in a mouse model for Alzheimer's disease, miR 106b was demonstrated to influence TGF-β signaling through the direct inhibition of the TGF-β type II receptor (TβR II) translation indicating TβR II as a functional target of miR-106b (16). Starting from these evidences we investigated the role of miR17-92 and miR 106b-25 clusters in neuroinflammation in a mouse model of multiple sclerosis, EAE, and found that the absence of 17-92 inhibits EAE development. We can hypothesize that single (17-92^−/−^) and double (miR-106b-25^−/−^miR-17-92^−/−^) knockout mice were protected from clinical signs of EAE due to the alterations of immune responses already described in literature, such as lymphoproliferation (10), antigen presentation (17), and Th17 differentiation (18), potentially affecting the induction of the experimental autoimmune disease *per sé*.

As expected, we found a significant decrease of GM-CSF level in the spinal cord of double knock out mice. GM-CSF is a well-known pro-inflammatory cytokine essential for development and progression of EAE (19, 20). GM-CSF reduction in double knock out mice, which are protected from EAE development, is in line with previous findings. Indeed, GM-CSF is required by CCR2^+^ monocytes to initiate tissue inflammation and the deletion of GM-CSF receptor in this cell subset induces EAE resistance (21). Moreover, overexpression of GM-CSF leads to the invasion and expansion of inflammatory myeloid cells into the brain and is sufficient to induce spontaneous CNS inflammatory disease (22). We found GM-CSF mRNA significantly upregulated in spinal cords of 106b-25^−/−^ EAE mice. GM-CSF is not an obvious target of miR 106b-25, thus this finding may be secondary to the immune dysregulation in these mice.

We show here, however, that also IL-17 and Th17 cells, which are known to drive neuroinflammation, are importantly reduced in mice lacking both miR17-92 and miR106b as compared to mice deleted only for miR106b or WT EAE mice. We therefore hypothesized that this reduction may inhibit effector functions in the target tissue, and constitute a more specific inhibition of neuroinflammation.

Pathological evaluation of spinal cord from EAE mice confirmed our clinical findings. We found that deletion of miR17-92 or both miR17-92 and miR106b was associated to a significant decrease of inflammatory infiltrates, demyelination, and axonal damage. Clinical and pathological analysis of EAE mice showed that there were no differences in miR 106b^−/−^ compared to wild type mice. Our RT-PCR data may suggest that this was due to function redundancy among miRNAs family clusters since miR17-92 and miR 106b-25 share the same set of target genes (10). While miR17-92 compensates for the absence of miR106b-25 in terms of increased expression, the opposite is not true.

Although we demonstrated a protective role for miR17-92 deletion we did not find the plausible targets of 17-92 that mediate this process. Quantification of the three major miR17-92 and miR 106b 25 targets (STAT 3, BIM, and P21) did not show any significant statistical difference in their levels among the different mice strains.

The study of Liu et al. (18) suggests a possible mechanism for miR17-92 cluster in Th17 differentiation. Indeed miR-19b and miR-17 enhances Th17 polarization by repressing the expression of Phosphatase and Tensin Homology (PTEN) and inhibiting Ikaros Family Zinc Finger 4 (IKZF4), respectively.

Our work underlines the relevance of the miR17-92 cluster for the development of experimental neuroinflammation, possibly for the inhibition of specific pathways, such as Th17 cells, crucial for EAE initiation. Indeed, we had found an imbalance of miR106b-25 and miR17-92 clusters also in human MS, suggesting that miR106b-25 and miR17-92 clusters may represent a plausible therapeutic target in human neuroinflammatory diseases.

## Materials and Methods

### Mice

All animal experiments were done with permission from the Institutional Animal Care and Use Committee (IACUC). All mice were maintained under specific pathogen-free conditions in the animal facility at San Raffaele Scientific Institute.

miR-106b-25, miR-17-92 KO, and miR-106b-25/miR-17-92 DKO mice were kindly provided by Dr. Paolo Dellabona (San Raffaele Scientific Institute, Italy) and generated as described by Ventura et al. (10) using 6- to 8-weeks-old C57BL/6 female mice purchased from Charles River Laboratories (Calco, Italy).

### Induction of Eae

C57BL/6 WT and mutant mice (8–10 weeks old) were immunized subcutaneously with 200 μg of MOG_35−55_ in Freund's Adjuvant (Sigma-Aldrich, St. Louis, MO, USA) supplemented with 4 mg/mL heat-killed Mycobacterium tuberculosis (strain H37Ra; Difco, Florence, Italy). Each mouse was injected i.v. with Pertussis toxin (500 ng, List Biological Laboratories, Campbell, CA, USA) on the day of the immunization and 48 h later. Mice were weighed and scored for clinical signs daily. Clinical symptoms of EAE were classified as follow: 0 = no signs; 1 = tail paralysis; 2 = ataxia and/or paresis of hindlimbs; 3 = paralysis of hindlimbs and/or paresis of forelimbs; 4 = tetraparalysis; and 5 = moribund or dead. EAE mice were killed at 37 d.p.i for real-time PCR, histological evaluation and flow cytometer analysis.

### Histological Evaluation

Pathological evaluation of spinal cord from EAE mice was performed 37 days post-EAE induction. Three mice per group were perfused through the left cardiac ventricle with saline plus EDTA 0.5 mM for 10 min followed by fixation with cold 4% paraformaldehyde, PFA (Sigma). Spinal cords from EAE mice were dissected out and post-fixed in 4% PFA overnight. Tissues were embedded in paraffin, sectioned and stained with Hematoxylin and Eosin, Kluver Barrera, and Bielschowsky to reveal perivascular inflammatory infiltrates, demyelinated areas, and axonal loss, respectively. Parameters were quantified on an average of 9 complete cross-sections of spinal cord per mouse taken at eight different levels. The number of inflammatory infiltrates were expressed as the number of infiltrates per mm^2^, demyelinated areas and axonal loss were expressed as percentage per mm^2^.

### RNA Extraction

Total RNA from splenic CD4+ T (isolated from mouse spleen with CD4+T cell isolation kit, Miltenyi Biotec GmbH) was isolated with the miRvana kit (Life Technologies). For mRNA extraction from brain, spinal cord, lymph nodes, and spleen tissues were homogenized with 1 ml of TRIzol (Life Technologies, Paysley, UK) every 50–100 mg using an IKA Ultra Turrax rotor homogenizer (Sigma Aldrich, St. Louis, MO, USA). RNA was quantified by Nanodrop ND 1000 spectrophotometer (Nanodrop Technologies Inc., Wilmington, DE, USA).

### Quantitative Real-Time Rt-Pcr

Reverse transcription of miRNA was performed using TaqMan MicroRNA Reverse Transcription kit (Applied Biosystems). qRT-PCR was performed with TaqMan MicroRNA Assay Mix containing PCR primers and TaqMan probes (Applied Biosystems). Values were normalized to snoRNA-202. The primers used in real-time RT-PCR experiments were as follows: miR 106b (Taqman Assay Applied Biosystems name: has-miR-106b; miRBase/Exiqon name: mmu-miR-106b; assay ID: 000442), miR 17 (has-miR-17; mmu-miR-17; 002308), miR 18a (has-miR-18a; mmu-miR-18a; 002422), miR 19a (has-miR-19a; mmu-miR-19a; 000395), miR 19b (has-miR-19b; mmu-miR-19b; 000396), miR 20a (has-miR-20a; mmu-miR-20; 000580), miR 25 (has-miR-25; mmu-miR-25; 000403), miR 92 (has-miR-92; mmu-miR-92a-3p; 000430), miR 93 (has-miR-93; mmu-miR-93; 001090), snoRNA-202 (assay ID: 001232).

For mRNA expression, RNA reverse transcription was performed with the High-Capacity cDNA Reverse Transcription kit (Applied Biosystems). STAT3 (Mm01219775 m1), BIM (Mm00437796 m1), p21 (Mm00432448 m1), GM-CSF (Mm00438328 m1), IFNγ (Mm01168134 m1), IL-17 (Mm00439618 m1) mRNA levels were measured by real-time RT-PCR using Taqman technology (Applied Biosystems, Invitrogen). PCR reactions were run on an ABI Prism 7500 Sequence Detection System. GADPH (4352339E) was used as a housekeeping gene. Relative changes in gene expression were determined using the 2^−ΔΔCT^ method.

### Flow Cytometry

Mouse-specific anti-CD3 (Pacific Blue conjugated, clone 500A2, catalog no. 558214), anti-CD4 (PerCP conjugated, clone RM4-5, catalog no. 553052), anti-CD25 (Phycoerythrin/Cy7, clone PC61, catalog no. 561780), anti-FoxP3 (Alexa Fluor 488 conjugated, clone MF23, catalog no. 560403), anti-IL17A (Alexa Fluor 647 conjugated, clone TC11-18H10, catalog no. 560184), and IFNγ (PE conjugated, clone XMG1.2, catalog no. 554412) mAbs were all purchased from BD Biosciences.

For intracellular staining total splenocytes were isolated 37 days after EAE induction and cells were re-stimulated for 3 days with increasing amounts of MOG35-55. After that cells were stimulated for 4 h with PMA (50 ng/ml) and ionomycin (500 ng/ml) in the presence of GolgiPlug (1:1,000, BD Pharmingen). Cell were stained with surface markers (CD3, CD4, CD25), permeabilized using the eBioscience™ Foxp3/Transcription Factor Staining Buffer Set and stained for IL-17, IFNg, and FoxP3.

Samples were acquired on BD FACS Canto II flow cytometer and analyzed with FlowJo software.

The absolute count of Treg, Th1, and Th17 cells was measured as % singlets on 20,000 cells X % Treg or Th1 or Th17 on singlets/100.

### Statistical Analyses

Differences between survival curves were calculated by Log-rank test (Mantel-Cox) while Mann-Whitney tests were used to evaluate differences between groups for non-parametric data. The results are expressed as means ± SEM. Differences are considered statistically significant when *p* < 0.05.

Statistical analyses were performed using GraphPad Prism version X (GraphPad Software, San Diego, CA, USA).

## Data Availability Statement

The datasets generated for this study are available on request to the corresponding author.

## Ethics Statement

This animal study was reviewed and approved by Institutional Animal Care and Use Committee (IACUC) and IRCCS Ospedale San Raffaele.

## Author Contributions

AF, MD, LC, CA, and GD performed the experiments. MF, PD, GC, and RF designed the experiments and analyzed the results. AM and RF wrote the manuscript. All authors contributed to the article and approved the submitted version.

## Conflict of Interest

The authors declare that the research was conducted in the absence of any commercial or financial relationships that could be construed as a potential conflict of interest.

## Supplementary Material

Figure S1 One representative image of the whole section of spinal cord presented in Figure 3. Spinal cord sections were stained with Hematoxylin and Eosin, Kluver Barrera, and Bielschowsky to asses number of infiltrates, demyelination, and axonal loss.

Figure S2 mRNA levels of classical targets for miR17-92 and miR106b. mRNA levels of p21, BIM, and STAT3 were measured in the brain, spinal cord, lymph nodes and spleen by real time RT-PCR. Data are shown as arbitrary units (AU). ^*^*P* < 0.05 (Mann Whitney test).
