# Supplementary figures and images for: Mir106b-25 and Mir17-92 Are Crucially Involved in the Development of Experimental Neuroinflammation
Source: Front Neurol. 2020 Aug 21;11:912. doi: 10.3389/fneur.2020.00912 (PMC7473303; doi:10.3389/fneur.2020.00912)

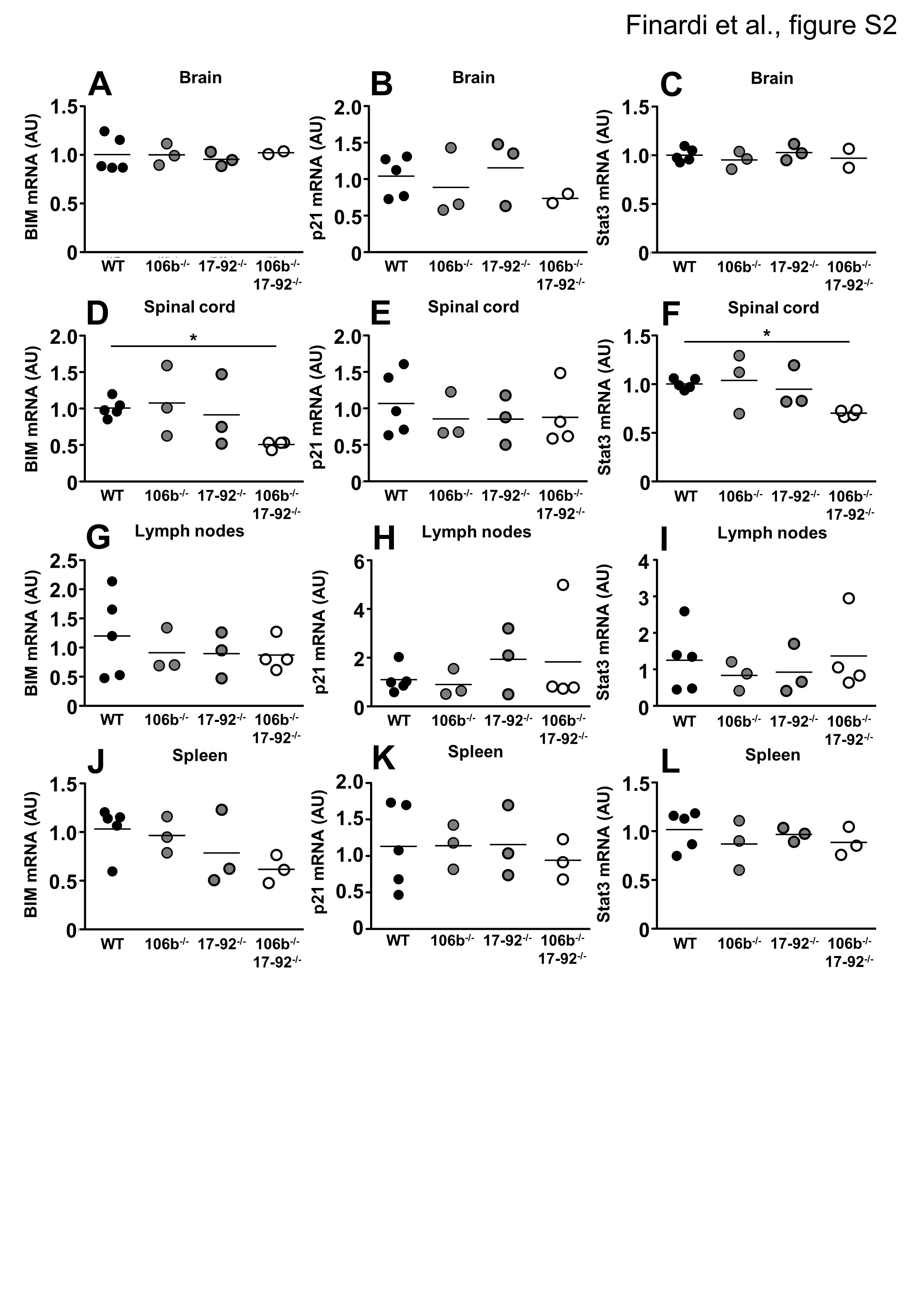

Supplement: Figure S1 — One representative image of the whole section of spinal cord presented in Figure 3. Spinal cord sections were stained with Hematoxylin and Eosin, Kluver Barrera, and Bielschowsky to asses number of infiltrates, demyelination, and axonal loss. [file Image_1.JPEG]

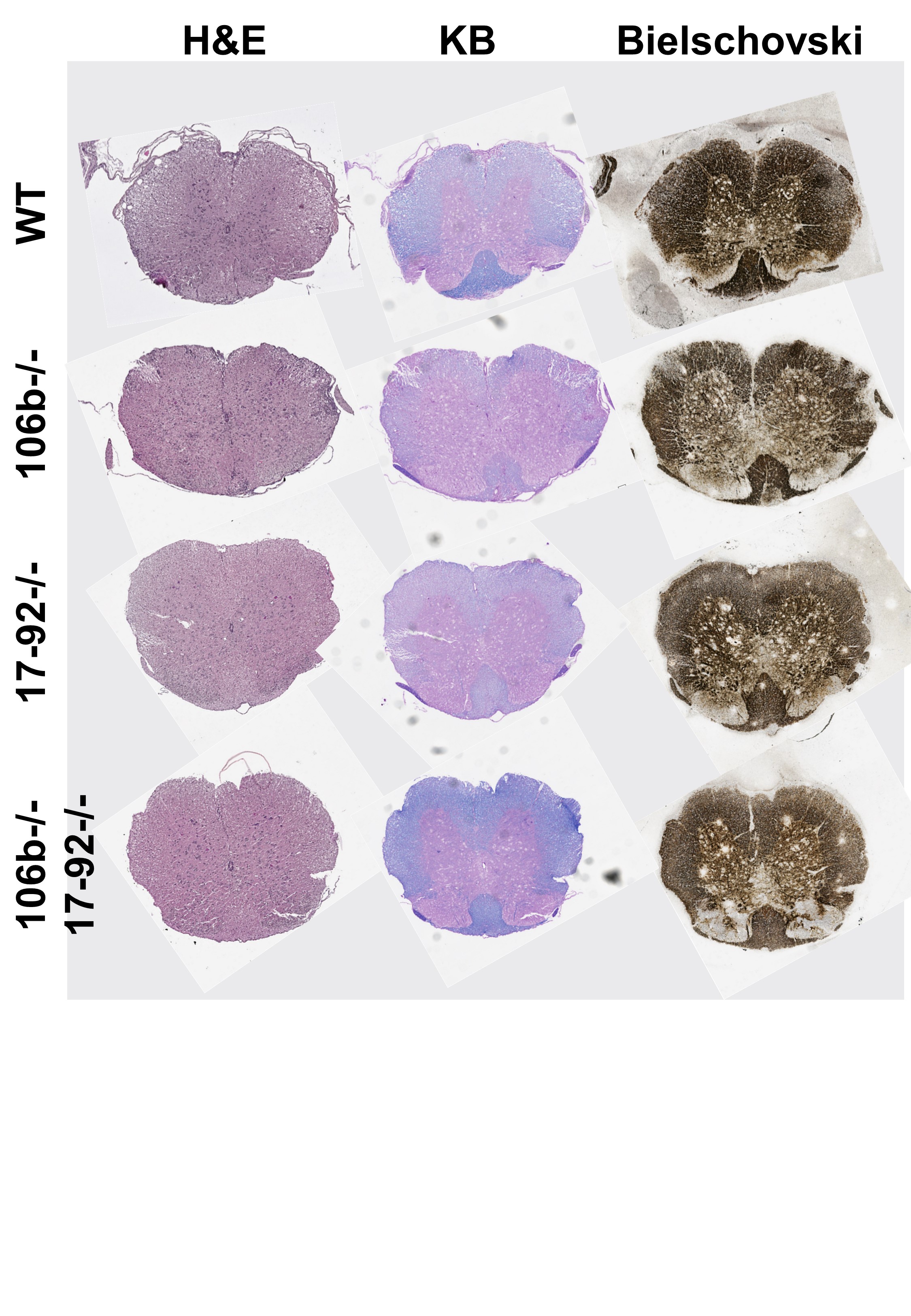

Supplement: Figure S2 — mRNA levels of classical targets for miR17-92 and miR106b. mRNA levels of p21, BIM, and STAT3 were measured in the brain, spinal cord, lymph nodes and spleen by real time RT-PCR. Data are shown as arbitrary units (AU). *P < 0.05 (Mann Whitney test). [file Image_2.JPEG]
